# Supplementary material for: Radiomics Based on Thyroid Ultrasound Can Predict Distant Metastasis of Follicular Thyroid Carcinoma
Source: J Clin Med. 2020 Jul 8;9(7):2156. doi: 10.3390/jcm9072156 (PMC7408789; doi:10.3390/jcm9072156)
Supplement: Supplementary file 1 [file jcm-09-02156-s001.pdf]

SUPPLEMENATARY MATERIAL

Table 1. Intraclass coefficient of each radiomics feature.

**Radiomics features (*n* = 60)**	**ICC**	**Radiomics features (*n* = 60)**	**ICC**
Firstorder_90Percentile	0.9850	GLCM_IDM	0.9379
Firstorder_Energy	0.9822	GLCM_IDMN	0.9173
Firstorder_Entropy	0.9036	GLCM_IDN	0.9295
Firstorder_InterquartileRange	0.9704	GLCM_IMC1	0.9691
Firstorder_Kurtosis	0.9504	GLCM_IMC2	0.9741
Firstorder_Maximum	0.8838	GLCM_InverseVariance	0.9755
Firstorder_MeanAbsoluteDeviation	0.9776	GLCM_JointAverage	0.9259
Firstorder_Mean	0.9912	GLCM_JointEnergy	0.9737
Firstorder_Median	0.9933	GLCM_JointEntropy	0.9152
**Firstorder_Minimum**	0.9939	GLCM_MCC	0.9755
Firstorder_Range	0.8792	GLCM_MaximumProbability	0.9736
Firstorder_RobustMeanAbsoluteDeviation	0.9771	GLCM_SumAverage	0.9259
Firstorder_RootMeanSquared	0.9888	GLCM_SumEntropy	0.9116
Firstorder_Skewness	0.9532	GLCM_SumSquares	0.8957
Firstorder_TotalEnergy	0.9822	GLSZM_GrayLevelNonUniformity	0.9732
Firstorder_Uniformity	0.9407	**GLSZM_GrayLevelNonUniformityNormalized**	0.8215
Firstorder_Variance	0.9705	GLSZM_GrayLevelVariance	0.8788
**Shape_Elongation**	0.9572	GLSZM_HighGrayLevelZoneEmphasis	0.8554
Shape_PerimeterSurfaceRatio	0.9746	GLSZM_LargeAreaEmphasis	0.9603
**Shape_Sphericity**	0.8062	GLSZM_LargeAreaHighGrayLevelEmphasis	0.8366
GLCM_Autocorrelation	0.8912	GLSZM_LargeAreaLowGrayLevelEmphasis	0.9889
GLCM_ClusterProminence	0.8465	GLSZM_LowGrayLevelZoneEmphasis	0.9625
GLCM_ClusterShade	0.9272	**GLSZM_SizeZoneNonUniformity**	0.9523
GLCM_ClusterTendency	0.8966	GLSZM_SizeZoneNonUniformityNormalized	0.9593
GLCM_Contrast	0.9174	GLSZM_SmallAreaEmphasis	0.9403
GLCM_Correlation	0.9759	GLSZM_SmallAreaHighGrayLevelEmphasis	0.8480
GLCM_DifferenceAverage	0.9285	**GLSZM_SmallAreaLowGrayLevelEmphasis**	0.9614
GLCM_DifferenceEntropy	0.9255	GLSZM_ZoneEntropy	0.9336
GLCM_DifferenceVariance	0.9148	GLSZM_ZonePercentage	0.9210
GLCM_ID	0.9411	GLSZM_ZoneVariance	0.9603

Note: GLCM=Gray Level Co-occurrence Matrix; ID=Inverse Difference; IDM=Inverse Difference Moment; IDMN= Inverse Difference Moment Normalized; IDN=Inverse Difference Normalized; IMC=Informational Measure of Correlation; MCC=Maximal Correlation Coefficient; GLSZM=Gray Level Size Zone Matrix; Features in bold denote selectee features.

TABLE 2. Correlation between selected radiomics features and important clinical variables. The first value in each cell element is *r*-value followed by *p*-value in the format of *r*-value (*p*-value).

Variables	Tumor Size	Echogenicity	Rim Calcification	Nodule-in-nodule appearance
Minimum	-0.034(0.661)	0.661(0.602)	-0.008(0.923)	0.072(0.351)
Elongation	0.055(0.474)	0.474(0.682)	0.010(0.899)	-0.016(0.833)
Sphericity	0.178(0.021)	0.021(0.514)	0.124(0.109)	-0.012(0.880)
Gray level non-uniformity normalized	-0.022(0.780)	0.780(0.987)	-0.036(0.641)	0.010(0.901)
Size zone nonuniformity	0.070(0.367)	0.367(0.376)	-0.025(0.747)	-0.047(0.541)
Small area low gray-level emphasis	-0.071(0.359)	0.359(0.962)	-0.003(0.969)	-0.069(0.372)

SUPPLEMENTARY MATERIAL. Software code for procedure (SVM code)

The code is available at https://github.com/skkuej/thyroid_SVM/blob/master/lasso_svm.mat.

```
%% data load, cross validation, z-normalization
X= data(:,3:62); % radiomics normalized_features(n=60),
y= data(:,2); % binary-metastases(n=169)
normalized_features = zscore(X(:,1:60));

foldMax = 5;
cvNum = 1;
c = cvpartition(y,'kfold',foldMax);

while cvNum <= foldMax
    trainingFeature = normalized_features(c.training(cvNum),:);
    testFeature = normalized_features(c.test(cvNum),:);
    trainingLabel = y(c.training(cvNum));
    testLabel = y(c.test(cvNum));

    %% Feature selection - Lasso
    lasso = cvglmnet(trainingFeature, trainingLabel, 'binomial');
    s(cvNum).selected_features = find(lasso.glmnet_fit.beta(:,(lasso.lambda == lasso.lambda_min)));
    trainingFeature = trainingFeature(:,s(cvNum).selectednormalized_features);
    testFeature = testFeature(:,s(cvNum).selectednormalized_features);

    %% SVM
    svmMdl = fitcsvm(trainingFeature,trainingLabel,'Prior','uniform');
    [labelHatTr, scoreTr] = svmMdl.predict(trainingFeature);
    [labelHatTs, scoreTs] = svmMdl.predict(testFeature);
    radiomics_score(cvNum).score = scoreTs;

    %% model evaluation
    [Xtr,Ytr,Ttr,AUCtr(cvNum)] = perfcurve(trainingLabel,scoreTr(:,2),1);
    [Xts,Yts,Tts,AUCts(cvNum)] = perfcurve(testLabel,scoreTs(:,2),1);
    conMat_train = confusionmat(trainingLabel, labelHatTr);
    ACC_train(cvNum) = (conMat_train(1,1)+conMat_train(2,2))/sum(conMat_train(:));
    SENS_train(cvNum) = conMat_train(2,2)/(conMat_train(2,1)+conMat_train(2,2));
    SPEC_train(cvNum) = conMat_train(1,1)/(conMat_train(1,1)+conMat_train(1,2));
    conMat_test = confusionmat(testLabel, labelHatTs);
    ACC_test(cvNum) = (conMat_test(1,1)+conMat_test(2,2))/sum(conMat_test(:));
    SENS_test(cvNum) = conMat_test(2,2)/(conMat_test(2,1)+conMat_test(2,2));
    SPEC_test(cvNum) = conMat_test(1,1)/(conMat_test(1,1)+conMat_test(1,2));
    cvNum = cvNum + 1;
end
```
